# Hyperuricemia, the heart, and the kidneys – to treat or not to treat?

**DOI:** 10.1080/0886022X.2020.1822185

**Published:** 2020-09-25

**Authors:** Tadej Petreski, Robert Ekart, Radovan Hojs, Sebastjan Bevc

**Affiliations:** a Department of Nephrology, Clinic for Internal Medicine, University Medical Centre Maribor, Maribor, Slovenia; b Faculty of Medicine, University of Maribor, Maribor, Slovenia; c Department of Dialysis, Clinic for Internal Medicine, University Medical Centre Maribor, Maribor, Slovenia

**Keywords:** Uric acid, asymptomatic hyperuricemia, chronic kidney disease, cardiovascular disease, hyperuricemia treatment

## Abstract

**Background:**

Hyperuricemia is a state in which the serum levels of uric acid are elevated. As such it has a pronounced effect on vascular and renal function with their consequences, while also showing some antioxidant effects that show to be beneficial.

**Summary:**

Hyperuricemia has shown to have a J-shaped relationship with mortality, is frequently associated with development and progression of heart and kidney disease, and is correlated with malnutrition-inflammation-atherosclerosis syndrome, although several Mendelian studies have failed to show an association with morbidity and mortality. Hyperuricemia is usually associated with gout flares and tophi development but can also present as asymptomatic hyperuricemia. It is still uncertain whether asymptomatic hyperuricemia is an independent risk factor for cardiovascular or renal disease and as such its treatment is questionable.

**Key messages:**

Some possible tools for future decision making are the use of noninvasive techniques such as pulse wave analysis, urinary sediment analysis, and joint ultrasound, which could help identify individuals with asymptomatic hyperuricemia that could benefit from urate lowering therapy most.

## 1. Introduction

Uric acid or urate is a molecule circulating in plasma. Some of it is ingested in the form of various purine rich foods, while some purines are endogenous: from synthesis or catabolism of nucleic acids. Nevertheless, purines are metabolized by xanthine dehydrogenase or xanthine oxidase to form uric acid [1,2]. Humans do not possess uricase, an additional enzyme in purine metabolism that is present in most mammals, so once urate is created it cannot be metabolized further but must be eliminated by the kidney, which accounts for about 70% of serum uric acid (SUA) excretion, or the intestines and its bacteria, which removes the remaining 30%. Herein lies the cause why hyperuricemia develops in 90% due to impaired renal excretion and why approximately 50% of chronic kidney disease (CKD) patients already become hyperuricemic prior to initiation of hemodialysis treatment [2–5]. To date, three urate transporters have been identified named urate transporter 1 (URAT1), glucose transporter 9 (GLUT9), and ATP binding cassette, subfamily G, 2 (ABCG2), and their dysfunction is the major cause of hyperuricemia. Additionally, hyperuricemia can also develop due to increased generation of uric acid [6]. On the other hand, the mechanism of extra-renal elimination is still poorly understood. Studies on mice revealed the breast cancer resistance protein (BCRP) transporter as one of the first transporters to contribute in part to the intestinal excretion, as its dysfunction increased SUA levels [7]. Recent reviews identified several more, such as, monocarboxylate co-transporter 9 (MCT9), Na^+^/phosphate co-transporters 4 and 5 (NPT4, NPT5), organic anion transporter 10 (OAT10), multidrug resistance proteins 2 and 4 (MRP2 and MRP4) [8].

Even though SUA is an end product of purine metabolism, it still has a biological effect, primarily affecting renal and vascular function. Experimental studies suggest that hyperuricemia causes kidney damage due to systemic and glomerular hypertension. One of several mechanisms suggested is inhibition of nitric oxide synthesis, which leads to arterial vasoconstriction. Another is induction of oxidative stress and inflammation [3,5,9–13]. On the other hand, urate is also an antioxidant with favorable effects, which are exhibited mostly in the central nervous system. It neutralizes glutamate and other free radicals, which might prove beneficial in acute stroke. Additionally, low values have been linked to neurodegenerative diseases such as Alzheimer’s disease, Huntington’s disease, Parkinson’s disease, and multiple sclerosis, which undoubtedly limit our SUA lowering possibilities [14,15]. Furthermore, lower SUA also suggests malnutrition and low protein intake presented as malnutrition-inflammation-atherosclerosis syndrome [15]. To summarize, SUA has shown to have a J-shaped relationship with all-cause mortality, meaning that low and high values were associated with death [16].

## 2. Hyperuricemia

One of the problems in dealing with hyperuricemia is no consensus on the definition of hyperuricemia. A practical value would seem to be SUA concentrations higher than 405 µmol/L (6.8 mg/dL) as this is urate’s solubility point measured using automated enzymatic methods in laboratories [10]. Other researchers suggest lower values down to 300 µmol/L (5 mg/dL) for healthy individuals, some suggest different values for men and women due to the uricosuric effect of estrogenic compounds, while others suggest values of 360 µmol/L (6 mg/dL) that are targeted when using urate lowering therapy (ULT) and is also the cutoff used in the latest recommendations by the European League Against Rheumatism (EULAR) [11,17–19].

On the other hand, asymptomatic hyperuricemia is a condition, where patients have elevated levels of SUA, yet do not exhibit symptoms or signs of monosodium urate crystal deposition, such as gout attacks, urolithiasis, or uric acid nephropathy. Campion et al. reported that during a follow up of 15 years, the annual incidence of gout for initially healthy men with their SUA between 420 and 470 µmol/L (7 and 7.9 mg/dL) was 0.5%. They concluded that most patients with hyperuricemia remain asymptomatic [20].

## 3. Treatment options for hyperuricemia

Effect of lifestyle and therapy on SUA levels is summarized in Table 1.

**Table 1.** Summary of foods, conditions, and drugs that affect SUA.

Raises SUA	Lowers SUA
Meat, seafood, soft drinks, fructose, honey, alcohol	Vitamin C, bing cherry, coffee, folic acid, low fat dairy
CKD	Weight loss and physical activity
OSAHS	
Acetylsalicylic acid, cyclosporine, theophylline, mycophenolate, beta- and alpha-1-adrenergic antagonists, ACE inhibitors	Lactobacillus^a^
	ULT
	Losartan, atorvastatin, calcium channel blockers, SGLT-2 inhibitors, fenofibrate, sevelamer, metformin, ARNI

SUA: serum uric acid; CKD: chronic kidney disease; OSAHS: obstructive sleep apnea–hypopnea syndrome; ACE: angiotensin-converting enzyme; ULT: urate lowering therapy; SGLT-2: sodium-glucose co-transporter 2; ARNI: angiotensin-II/neprilysin inhibitors.

^a^ Lowers SUA in rats.

### 3.1. Diet and lifestyle

The first line of hyperuricemia treatment is undoubtably using a low-purine diet, which can reduce SUA levels by 10–15%. It is wise to avoid meat, sea foods, soft drinks, fructose rich beverages, honey, and alcohol. Some agents have also proven to be effective in lowering SUA, such as vitamin C, so diets rich in vegetables and fruit can be helpful. Furthermore, bing cherry, coffee, low-fat dairy products, and folic acid have shown to be protective. Additionally, weight loss and physical activity are recommended [9,11,17].

A study on Bangladeshi adults has reported a significant positive relationship between SUA and obesity [21]. Furthermore, Dong et al. have observed an association between visceral adiposity and hyperuricemia independent of metabolic health and obesity phenotypes [22]. These findings are supported by a Mendelian randomization study as well. They reported an increase in SUA of 0.3 mg/dL and gout odds ratio of 2.24 for each increase in genetically predicted BMI of 4.6 kg/m^2^ [23]. Another factor that has shown an association with higher SUA is obstructive sleep apnea–hypopnea syndrome (OSAHS) [24]; however, meta-analyses have not revealed an effect of using continuous positive airway pressure (CPAP) in OSAHS on SUA levels [25].

Recently, studies on mice have shown that probiotics could reveal to be an additional therapeutic option. *Lactobacillus* strains have shown to lower SUA with beneficial effects on hypertension and renal disease [26,27].

### 3.2. Urate lowering therapy

The main pharmacologic agents used for ULT are xanthine oxidoreductase (XOR) inhibitors. XOR inhibitors reduce SUA levels by inhibiting uric acid synthesis [28]. Allopurinol is a purine XOR inhibitor with an active metabolite oxypurinol, which acts as a reversible covalent inhibitor and is eliminated by the kidneys. Febuxostat on the other hand is a highly potent non-purine noncompetitive XOR inhibitor that undergoes hepatic metabolism. Recently, topiroxostat, a novel hybrid-type inhibitor has been approved, but further studies are needed [28,29]. Both allopurinol and febuxostat are generally safe drugs but can have serious adverse effects in a small percent of users, thus it would deem appropriate to try to use the lowest values of allopurinol possible, such as 100 mg daily, especially in CKD patients, which might have the same outcome as higher values, but with lower risk. An additional effect to lowering urate is inhibition of reactive oxygen species formation, which may also have beneficial renal effects [5,15]. Some studies have shown that in patients with CKD and hyperuricemia resistant to allopurinol treatment, febuxostat was well tolerated and managed to lower SUA levels in most patients [30]. However, recently a noninferiority trial was conducted comparing febuxostat to allopurinol in 6190 patients with gout, where they observed a higher mortality in the febuxostat group [31].

Other possible ULT agents are uricosurics such as probenecid, benzbromarone, lesinurad and recently a selective urate reabsorption inhibitor (SURI) named dotinurad [32,33]. Current evidence points to underexcretion as the main cause of hyperuricemia, compared to overproduction, which is why more thought has been put into development of SURIs, while clinical trials evaluating their efficacy and safety are warranted [33]. When using uricosurics, urine should be alkalinized to prevent formation of kidney stones. Benzbromarone was withdrawn from the European market due to some reports of hepatotoxicity but has stayed available in several other countries. It inhibits the apical urate exchanger in proximal tubules, which reduces urate reabsorption and lowers serum levels. Additionally, uricosurics are contraindicated when an estimated glomerular filtration rate (eGFR)<50 mL/min, which limits their use in CKD, although some studies have been done for testing their efficacy and safety profile [32–37]. Recently, a novel agent called pegloticase, a pegylated recombinant mammalian urate oxidase, has been developed. Due to its high potency, loss of treatment responsiveness due to antibody development, and infusion related risks, it is recommended only for refractory gout [38]. A review published in 2018 has listed and covered 11 drugs to treat hyperuricemia in active development [39].

The 2016 EULAR recommendations on management of gout recommend starting treatment with allopurinol. If target SUA cannot be reached, they recommend use of febuxostat, a uricosuric or a combination of a uricosuric with a xanthine oxidase inhibitor. Pegloticase is recommended for refractory gout [40].

### 3.3. Existent therapy modification

Another option for treating hyperuricemia is the use of medications with secondary SUA lowering capabilities such as losartan, atorvastatin, calcium channel blockers, sodium-glucose co-transporter 2 (SGLT-2) inhibitors, fenofibrate, sevelamer, metformin, and angiotensin-II/neprilysin inhibitors (ARNI). Some medications that should be considered for discontinuation because of their known effect to raise SUA are acetylsalicylic acid, cyclosporine, theophylline, mycophenolate, beta- and alpha-1-adrenergic antagonists, and angiotensin-converting enzyme (ACE) inhibitors [5,15,41–44].

Interesting data were found regarding medications with urate lowering capabilities as a secondary effect. Use of losartan has decreased the chance of renal disease by 6% for every 0.5 mg/dL decrease in SUA levels, which was also observed in a *post hoc* analysis on diabetes mellitus (DM) patients [5]. Several other studies have also found losartan to be exceptional amongst antihypertensives in possessing mild uricosuric properties [17,31]. An additional SUA lowering effect was also described in patients treated with a combination of amlodipine and losartan, where they reached a 1.9 mg/dL decrease in SUA after 3 months. Similar effects were witnessed in several other studies [34,45].

Regarding the use of statins, a meta-analysis showed urate lowering effects observed only with use of atorvastatin and simvastatin, but not others [46]. With atorvastatin some researchers have also observed a raise in eGFR [47]. Fenofibrate was found exhibiting similar urate lowering properties, so treatment with these lipid lowering agents could prove especially beneficial in patients with a higher coronary event risk and hyperuricemia [48].

Lastly, SGLT-2 inhibitors have been observed to lower SUA. A meta-analysis has shown empagliflozin to be superior to others in this regard and better results were achieved during early DM. However, the effect was abolished in patients with CKD [49]. In comparison with glucagon-like peptide-1 (GLP-1) receptor antagonists, SGLT-2 had lower incidence rate of gout [50].

## 4. Treatment of asymptomatic hyperuricemia

Currently, there is still no consensus whether to pharmacologically treat asymptomatic hyperuricemia. Some have suggested ULT in cardiovascular (CV) prevention strategies, but no official recommendations have been made. Pharmacotherapy is warranted only in patients receiving radiotherapy or chemotherapy, patients with hereditary enzyme deficiencies, or in patients with extreme values of SUA [9,19]. Nevertheless, asymptomatic hyperuricemia is actively treated in some countries like Japan to prevent non-gout disease such as arterial hypertension (AH), coronary events, and CKD. During 2010–2014, this resulted in a significant increase of asymptomatic hyperuricemia treatment from 1.8 to 2.1% of all insured patients in Japan [51]. Additionally, 85% of Japanese nephrologists are in favor of ULT for asymptomatic hyperuricemia in patients with CKD stage 3–5 [52]. Furthermore, during a 5-year follow up of Japanese patients, one study showed that hyperuricemia was linked with a statistically significant increase in incidence rates of AH, dyslipidemia, CKD, and weight gain [53]. Recently, a systematic review has found evidence that asymptomatic hyperuricemia should be treated only under specific circumstances: first, in patients with persistent SUA levels higher than 13 mg/dL in men or 10 mg/dL in women; second, when urinary excretion of uric acid exceeds 1100 mg daily; lastly, before initiation of radiation or chemotherapy [41].

## 5. Hyperuricemia and cardiovascular disease

It is still uncertain whether asymptomatic hyperuricemia is an independent risk factor for CV disease. In several epidemiological and observational studies, hyperuricemia has been linked to CV events, AH, and DM, but Mendelian randomization studies do not seem to show an association [18,54–58]. However, in healthy patients free of comorbidities, asymptomatic hyperuricemia has predicted the risk of incident CV events [59]. Additional evidence for the CV burden of hyperuricemia was also a study that showed SUA was independently associated with elevated coronary artery calcium scores [60]. Meta-analyses have shown that hyperuricemia is independently connected with an increased risk for incident AH [9,19]. Recently, Pilemann-Lyberg et al. have concluded, that higher SUA levels in patients with DM type 1 are associated with a higher risk of CV events, mortality, and kidney function decline, independently of other factors [61]. A recent review has additionally highlighted the importance of SUA on development and clinical prognosis of heart failure, where drugs that are associated with improved CV outcomes such as SGLT2 inhibitors and ARNI, also presented an SUA lowering capability [62].

Allopurinol use was associated with only a small reduction in blood pressure in adults of 3.3 mmHg systolic and 1.3 mmHg diastolic, while febuxostat has managed to lower systolic pressure in CKD patients for 7.2 mmHg and diastolic pressure for 5.1 mmHg [15,63]. However, reduction in blood pressure has not shown clinical benefit in patients with heart failure [9]. Recently, Johnson et al. have reported using pegloticase, a recombinant uricase, in patients with chronic gout refractory to standard therapy. They achieved a reduction in mean arterial pressure of 5 mmHg, independent of changes in renal function [64]. A meta-analysis has shown that allopurinol therapy is associated with improved endothelial function, which is more pronounced in patients with normal SUA at baseline [65]. Similar results were found in a systematic review, where allopurinol use, with its antioxidant properties, had a significant benefit on endothelial dysfunction in patients with congestive heart failure and in patients with CKD, but not type 2 DM [66]. It seems that allopurinol benefit is independent of administered dose, duration of treatment, or its effect on SUA levels [67]. Using febuxostat therapy resulted in similar effects on endothelial function in patients on hemodialysis treatment, with a significant drop in SUA levels [68,69]. Recently, a meta-analysis of randomized controlled trials (RCTs) on use of febuxostat carried out by Chewcharat et al. reported a reduction in systolic and diastolic blood pressure compared to placebo, apparently with no effect on major CV events, arrhythmias, or stroke events [70]. A systematic review on use of xanthine oxidase inhibitors for prevention of CV events has concluded that they may reduce the incidence of adverse CV outcomes when used in lower doses (<300 mg/day) [71].

The ongoing URRAH (Uric Acid Right for Heart Health) project has identified several SUA cutoff values for prediction of total mortality (4.7 mg/dL), CV mortality (5.6 mg/dL), and fatal myocardial infarction (5.7 mg/dL) [72].

## 6. Hyperuricemia and kidney disease

Patients with known gout have a twofold risk for developing kidney stones compared to healthy individuals. However, it remains unclear whether hyperuricemia is an independent risk factor for incident kidney stones, even though some evidence is revealing an association [73]. Epidemiologic studies have shown that the risk of incident CKD is twice higher in hyperuricemic patients regardless of DM presence [5,74]. Evidence supporting a causal link is also the development of renal disease in patients with familial juvenile hyperuricemic nephropathy, where hyperuricemia precedes renal disease [19]. Some studies have shown that hyperuricemia predicts progression of kidney disease in various nephropathies, especially in patients with IgA and diabetic nephropathy while others have shown no effect of hyperuricemia on progression of CKD [5,10,19]. A longitudinal analysis done on Taiwanese patients has shown that the risk of progression to renal failure increased 7% for each 60 µmol/L (1 mg/dL) increase in SUA and the effects were more prominent in patients without proteinuria [75]. Higher values of SUA and body mass index have been associated with eGFR decline of ≥50% or end-stage kidney disease in patients with arterial/arteriolar nephrosclerosis [76]. Furthermore, increased SUA correlates with increased intima media thickness values as markers of atherosclerosis and suboptimal blood pressure control [77]. Contrarily, some researchers have shown that hyperuricemia and higher SUA were associated with lower risk of all-cause and CV mortality in the hemodialysis population [78,79]. Additionally, a Mendelian randomization study found no evidence for a causal link between hyperuricemia and DM [19]. In the studies carried out in our center, we have shown that hyperuricemia is directly associated with higher all-cause and CV mortality of CKD patients [80,81].

Several meta-analyses have been carried out to test the effect of using ULT on CKD progression. A recent meta-analysis regarding use of allopurinol has shown a mean difference in lower eGFR decline of 3.2 mL/min/1.73 m^2^ compared to placebo, which was observed in other meta-analyses on CKD patients as well [15]. A study published recently by Vargas-Santos et al. showed that allopurinol initiation of at least 300 mg/day in newly diagnosed gout was associated with a lower risk of renal function decline [82]. Similar data were also published regarding the use of febuxostat, with additional effects on lowering blood pressure, while some researchers witnessed no effect of febuxostat on eGFR in CKD stage 3 patients with asymptomatic hyperuricemia [63,83–88]. Others showed that allopurinol is associated with a reduction of serum creatinine, with no effect on eGFR [5]. Recently, Kojima et al. reported in the FREED study that febuxostat effectively lowers uric acid to target values and delays the progression of renal dysfunction [88]. The aforementioned meta-analysis by Chewcharat et al. reported slowing of renal function decline regardless of baseline function in patients receiving febuxostat compared to placebo [70]. A head to head comparison (allopurinol vs. febuxostat) described higher efficacy of febuxostat to reach target values of SUA than allopurinol in six months and has also shown an increase in eGFR, while they observed a decline of eGFR in the allopurinol group [85]. All meta-analyses concluded that currently there is insufficient evidence to support the use of ULT for treatment of CKD progression [15,83]. However, a recent study showed that CKD patients on febuxostat followed for approximately 4.5 years had a significantly slower renal disease progression than patients on allopurinol or conventional CKD treatment regardless of potent cofounders. Additionally, only patients with febuxostat managed to reach target SUA levels of less than 360 µmol/L (6 mg/dL) [89]. Patients that are switched from allopurinol to febuxostat treatment also show similar results [90]. Additionally, a pilot study in patients with CKD stage 3 and 4 was done, comparing use of febuxostat against benzbromarone. They concluded that both agents could reduce SUA levels, maintain renal function and potentially improve anemia [36]. Chou et al. have compared both agents against allopurinol in CKD population and concluded, that febuxostat and benzbromarone could be more effective in lowering SUA levels and reducing the risk of progression to dialysis than allopurinol [37].

Recently, and open label study was published evaluating anti-albuminuric effects of topiroxostat in patients with diabetic nephropathy, where treatment with high dose (160 mg) topiroxostat reduced albuminuria compared to low dose (40 mg) [91].

## 7. Future assessment options

Researchers have suggested the use of urinary sediment analysis for the presence of urate crystals, which indicates supra-saturation values in the kidney, and musculoskeletal ultrasound for identification of urate deposition on joints in patients with asymptomatic hyperuricemia. Studies should evaluate the cost–benefit of testing to reveal patients that could benefit most and guide clinical practice. Others have suggested ultrasound of the first metatarsal joint in patients with family history of gout. ULT should be initiated and SUA followed-up, if pathologic findings are present [19,38]. A pilot study has shown that the presence of double contour sign (DCS) and hyperechoic cloudy area (HCA) on joint ultrasound provides a high sensitivity (100%) and specificity (88.2%) for detecting urate crystals in the synovium, which the EULAR recommendations perceive as a definitive diagnosis of gout [92]. If patients with gout are treated with ULT and achieve a goal target SUA levels of less than 360 µmol/L (6 mg/dL) for more than 6 months, urate crystals on joint cartilage will also disappear [93,94].

As atherosclerosis is one of the main causes of CV morbidity and mortality, and could be associated with hyperuricemia in all patients, future research in this area could also benefit from use of noninvasive tools for CV assessment such as pulse wave analysis and pulse wave velocity. A study has shown that higher SUA was associated with a greater increase in pulse wave velocity, pointing toward arterial stiffness in patients with SUA ≥ 370 µmol/L (6.2 mg/dL), which is more frequently reached in men [11,95]. Novel tools should be sought to test changes in SUA after initiation of therapy to evaluate their effect on CV and kidney function.

According to the review from Pascart and Richette [39], several medications with SUA lowering properties are emerging. Controlled randomized trials should be done to assess efficacy and safety, while keeping in mind to assess the effect of lowering SUA on kidney and heart disease.

## 8. Conclusions

Even though the topic of hyperuricemia is as old as most medications used for its treatment, controversy remains. As we wait for further trials to complete and give us a definitive answer whether we should start treatment of asymptomatic hyperuricemia, further thought should be given into individualizing patient treatment and substituting certain medications with others that have a similar primary end goal with additional effects regarding lowering of SUA. Time will also tell whether countries that have already begun with a more aggressive approach toward treatment will show better outcomes for patients, in the meantime use of musculoskeletal ultrasound and urinary analysis might guide the rest of us.
